# Risk Factors and Successful Interventions for Cricket-Related Low Back Pain: An Updated Systematic Review

**DOI:** 10.7759/cureus.79869

**Published:** 2025-03-01

**Authors:** Shwetabh Singh, James Baker, Stuart Egginton

**Affiliations:** 1 Sports Medicine, Hull Kingston Rovers, Hull, GBR; 2 Emergency Medicine, Bankstown-Lidcombe Hospital, Sydney, AUS; 3 School of Biomedical Sciences, Leeds University, Leeds, GBR

**Keywords:** biomechanics, bowling technique, cricket, injury prevention, lower back pain, lumbar spine, sports medicine

## Abstract

Lower back pain (LBP) poses a significant challenge for cricketers of all standards, often leading to rehabilitation periods exceeding eight months and potential termination of the playing season. Despite the identification of modifiable risk factors and interventions in previous studies, a comprehensive review of the past decade is absent. With the introduction of shorter formats like Twenty20 (T20) and the rise of franchise cricket, an updated evaluation of risk factors and interventions for preventing and treating LBP in cricketers is needed. This study critically assesses and summarises current understanding in this area, incorporating previous recommendations and considering the evolving cricket landscape.

A systematic review was conducted using databases such as SportsDiscus, MEDLINE, CINAHL, ISI Web of Knowledge, and Cochrane Library. Key terms related to LBP in cricketers were utilised. The Down and Black quality assessment tool, in addition to van Tulder's criteria for levels of evidence, was applied. The quantitative analysis involved meta-analyses conducted using IBM SPSS Statistics for Windows, Version 29 (Released 2023; IBM Corp., Armonk, New York).

Sixteen studies, of which 15 were of high quality, investigated risk factors associated with LBP. One low-quality randomised controlled trial examined LBP treatment. The meta-analysis revealed significant associations between LBP and increased workload, decreased bone mineral density, and poor lumbo-pelvic control through increased side flexion during the bowling action. Strong evidence supported the association between the presence of bone marrow oedema (BMO) and LBP.

Bone marrow oedema on magnetic resonance imaging (MRI) provides an early indicator before the development of stress fractures, serving as a valid and reliable screening tool. Workload monitoring plays a crucial role in identifying high-risk bowlers. However, further research is needed to establish causal relationships among several other risk factors outlined. Additionally, addressing the scarcity of high-quality interventional studies is of utmost importance.

## Introduction and background

**Figure 1:**
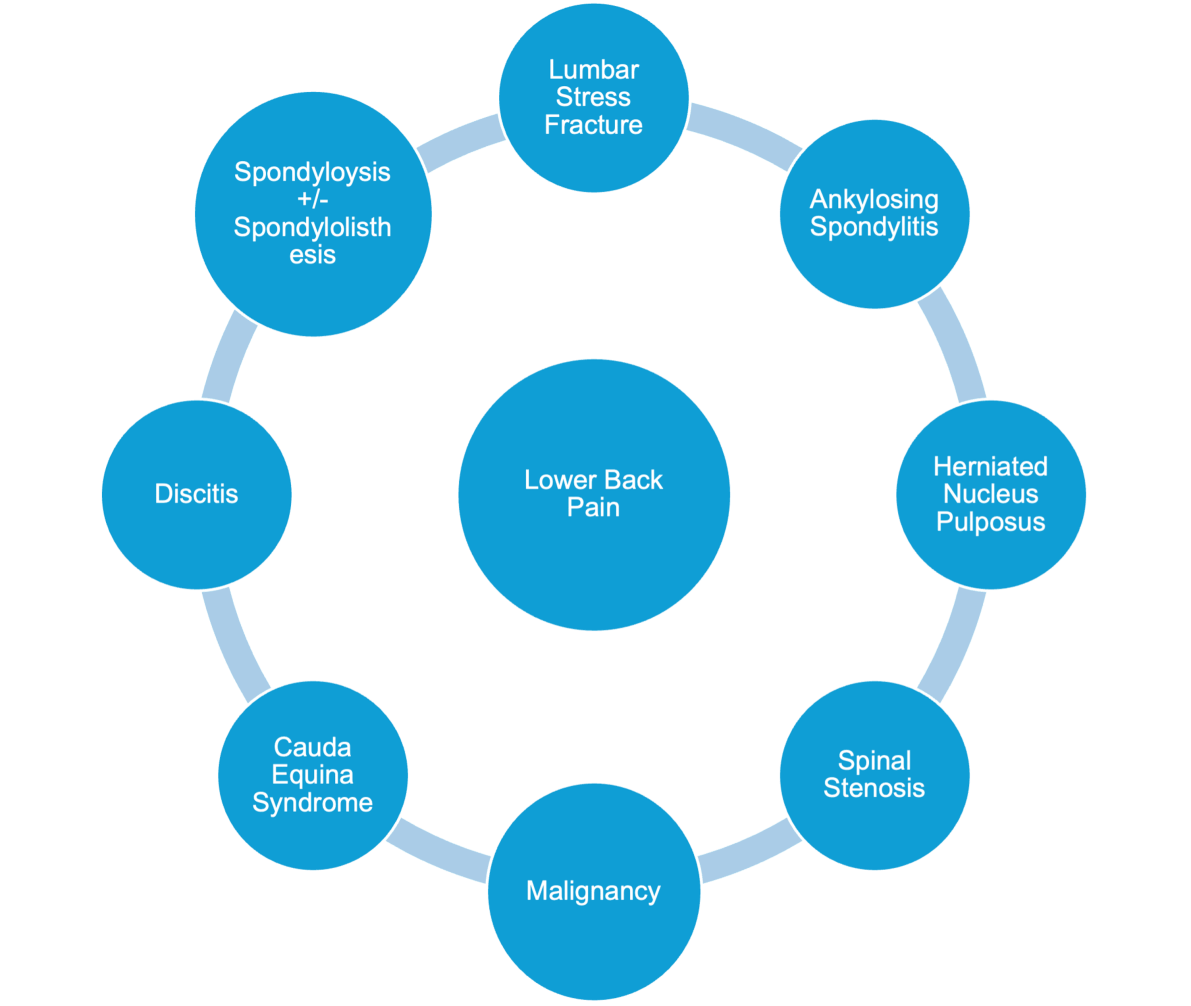
Summary of the differential diagnoses for LBP The image was created by the authors of this paper.

Cricket is a globally played sport that demands a combination of skill, endurance, and physical resilience. While often perceived as a non-contact sport, cricket places substantial physical demands on its players, particularly bowlers, who are exposed to repetitive high-impact forces. Among the most prevalent issues faced by cricketers is lower back pain (LBP), a condition affecting players across all levels, genders, and age groups. LBP is a broad term encompassing various spinal conditions, ranging from muscle strains to stress fractures, and has significant implications for player availability and career longevity (Figure 1) [1-3].

Although cricket does not involve direct physical collisions like rugby or football, injury rates remain notably high. Studies indicate that 20.6% of elite cricketers experience injuries severe enough to prevent selection, surpassing rates in professional football (20%) and rugby (12%) [4-6]. Among these injuries, lumbar stress fractures (LSF) are particularly concerning, with a prevalence of 24%-55% in adult fast bowlers and an even higher rate of 64% among junior bowlers [6-9]. These injuries can sideline players for extended periods, with recovery timelines often exceeding eight months [10]. Furthermore, elite women’s cricket reports 1.7 lumbar injuries per 100 match days, accounting for 25% of total lost match time over a four-year period [11-14].

The evolution of cricket in recent decades has further heightened injury risks. The introduction of shorter formats, such as Twenty20 (T20) and franchise leagues, has led to a 30% increase in match days globally, intensifying the physical burden on players [15]. Injury data show that match injury incidence is higher in limited-overs formats, with One Day Internationals (ODIs) and T20s averaging 263 and 194 lost match days per season, respectively, compared to 118 days in Test cricket [4]. These increased workloads contribute significantly to LBP risk, necessitating an updated understanding of injury mechanisms and preventive strategies.

Multiple studies have identified intrinsic and extrinsic factors that increase the likelihood of LBP in cricketers [16]. Intrinsic factors include biomechanical inefficiencies, such as improper bowling techniques that overload the lumbar spine, leading to stress fractures and soft tissue injuries [17-19]. Fast bowlers, in particular, are vulnerable due to the high forces generated during their delivery stride, with excessive side flexion and lumbar counter-rotation significantly elevating the risk of LBP [16, 20-22]. Muscle imbalances and asymmetry, particularly in the core and lumbar region, also contribute to spinal instability and increased susceptibility to injury [16, 20, 22, 23].

Conversely, extrinsic factors relate to training loads, playing conditions, and recovery practices. Overuse injuries are common in cricket due to repetitive bowling actions and inadequate recovery periods, with research highlighting a strong correlation between excessive match workloads and lumbar stress injuries [4, 24]. Surface conditions and equipment choices (e.g., footwear and bowling technique adaptations) further modulate injury risk [25, 26]. Additionally, younger players undergoing growth spurts face an increased risk of spinal stress injuries, while cricketers with prior back injuries are significantly more prone to recurrence [14, 27-29].

Research has focused extensively on biomechanics and workload monitoring as key determinants of LBP risk, especially in fast bowlers. Studies suggest that adolescents with excessive shoulder counter-rotation and adults with increased contralateral lumbar side flexion are particularly prone to stress fractures [30]. Systematic reviews have examined how neuromuscular imbalances, reduced trunk endurance, and impaired lumbo-pelvic-hip control contribute to LBP [21, 25, 30]. Additionally, magnetic resonance imaging (MRI) has been increasingly used to detect bone marrow oedema (BMO), an early indicator of lumbar bone stress injuries, before fractures develop [31]. These findings reinforce the importance of early screening and targeted biomechanical interventions to prevent LBP in cricketers [21].

Despite the wealth of research on cricket-related LBP, significant gaps remain in our understanding of injury mechanisms and intervention effectiveness. The last comprehensive review on this topic, conducted in 2012, highlighted a lack of high-quality studies and difficulties in establishing causal relationships between risk factors and injury outcomes [16]. However, with modern advancements in imaging, biomechanics, and workload monitoring, a reassessment of LBP risk factors and treatment strategies is urgently needed [31].

Given the evolving nature of cricket, marked by new playing formats, increased workload demands, and improved athlete conditioning, it is essential to reassess LBP risk factors and prevention strategies. This review aims to (1) systematically examine the latest evidence on risk factors contributing to LBP in cricketers, (2) evaluate interventions aimed at preventing and treating LBP, incorporating findings from the past decade, and (3) identify research gaps to inform future investigations and improve clinical recommendations for LBP management in cricket.

## Review

Materials and methods

This study refines the methodology of Morton et al. [16] to primarily investigate the aetiology of LBP in cricketers and secondarily to evaluate the impact of recent literature on the management and prevention strategies for LBP. Emphasising contemporary relevance, studies critiqued by Morton et al. [16] were excluded due to either substandard quality or obsolescence in the context of modern cricket. This strategic incorporation is used to assess advancements in LBP research, particularly in identifying novel diagnostic, risk, and intervention strategies. It ensures that findings and recommendations reflect the latest scientific insights and practical developments in cricket.

Literature Search

The following databases were utilised: SportsDiscus, MEDLINE, CINAHL, ISI Web of Knowledge, and Cochrane Library. The inclusion of studies considered those published from 20 November 2012 (the latest date covered by Morton et al. [16]) to 22 October 2022. Table 1 provides an overview of the inclusion/exclusion criteria applied to select studies for the review. To facilitate categorisation, interventions pertaining to the treatment and prevention of LBP in cricketers, as well as factors associated with LBP development, were assigned specific Medical Subject Heading (MeSH) terminology (Table 2). The process of data extraction adhered to the Preferred Reporting Items for Systematic Reviews and Meta-Analyses (PRISMA) guidelines [32]. EndNote software was used for this process. This study was registered with PROSPERO under the registration number CRD42023393147.

**Table 1:** Inclusion and exclusion criteria Inclusion and exclusion criteria for studies of risk factors and interventions for LBP in cricketers. LBP: lower back pain.

Studies of risk factors influencing LBP in cricketers: inclusion criteria	Studies of risk factors influencing LBP in cricketers: exclusion criteria	Studies of interventions for LBP in cricketers: inclusion criteria	Studies of interventions for LBP in cricketers: exclusion criteria
All the different roles of cricketers	Studies looking at extrinsic factors affecting LBP in cricketers outside of cricket (e.g., occupation)	All the different roles of cricketers	Patents
All different levels/standards of cricket	Studies researching athletes in sports that are not cricket	All different levels/standards of cricket	Conference/congress proceeding/letters
All ages	Outcome measure which is not relevant to LBP Patents	All ages	Observational design studies
All genders	Magazines	All genders	Government documents
English Language only	Conference/congress proceeding/letters Books Government Documents Reviews Theses Editorials	Intervention studies, which aim to treat and/or prevent LBP in cricketers	Books
Outcome measure to be relevant to LBP		English Language only	Magazines
Intrinsic factors linked to LBP (e.g., bowling technique/workload)			Reviews
			Theses
			Editorials

**Table 2:** MeSH headings LBP: lower back pain, MeSH: medical subject headings.

Area of study	MeSH search terms used
Risk factors of LBP in cricketers	“lumbar back pain” OR “back pain” OR “side strain” OR “back injury” OR “spine injury” OR “side injury” OR “muscle pain” OR “back injuries” OR “trunk injury” OR “trunk injuries” OR “trunk” OR “spine pathology” OR “spinal pathology” OR “injury” OR “spondylolysis” OR “disc degeneration” OR “spinal abnormalities” OR “lumbar” OR “spinal shrinkage” AND “cricket* OR “cricketers” OR “bowlers” OR “fast bowler” OR “spin bowler” OR “fast bowling” OR “bowling” AND “strength” OR “range of motion” OR “core stability” OR “ROM” OR “kinematics” OR “muscle size” OR “muscle asymmetry” OR “lumbar back pain” OR “back pain” OR “side strain” OR “back injury” OR “spine injury” OR “side injury” OR “muscle pain” OR “back injuries” OR “trunk injury” OR “trunk injuries” OR “trunk” OR “spine pathology” OR “spinal pathology” OR “injury” OR “spondylolysis” OR “disc degeneration” OR “spinal abnormalities” OR “lumbar” OR “spinal shrinkage”
Interventional studies for LBP in cricketers	“lumbar back pain” OR “back pain” OR “side strain” OR “back injury” OR “spine injury” OR “side injury” OR “muscle pain” OR “back injuries” OR “trunk injury” OR “trunk injuries” OR trunk OR “spine pathology” OR “spinal pathology” OR “injury” OR “spondylolysis” OR “disc degeneration” OR “spinal abnormalities” OR “lumbar” OR “spinal shrinkage” AND “cricket” OR “cricketers” OR “bowlers” OR “fast bowler” OR “spin bowler” OR “fast bowling” OR “bowling” AND “prevention” OR “intervention” OR “training” OR “technique” OR “strapping” OR “exercise” OR “injection” OR “rest” OR “coaching” OR “tape” OR “strengthen” OR “stability” OR “treatment?”

Quality Assessment

A modified version of Downs and Black's method was employed to evaluate risk factors associated with LBP, whilst the full checklist was utilised for interventional studies [16, 33, 34]. Question 27 was modified to inquire whether the study had sufficient power, with one point awarded if a significant difference was detected or if a sample size calculation was conducted. Studies exploring potential risk factors or factors linked to LBP that achieved a score of 10 or higher, and interventional studies that achieved a score of 20 or higher, were deemed high quality [16, 33, 34] (Appendices).

Data Extraction and Analysis

The relevant outcome measures were extracted from studies included in the review. Continuous data were used to calculate means and standard deviations (SD), which were then used to generate forest plots for meta-analysis. IBM SPSS Statistics for Windows, Version 29 (Released 2023; IBM Corp., Armonk, New York) was used to analyse data. In cases where non-parametric results were provided in the papers [28, 35, 36], the median and interquartile range (IQR) were converted to facilitate inclusion in the meta-analysis [37]. Attempts were made to contact the authors to obtain additional data for inclusion in the quantitative analysis. Some data from certain studies were not retrievable [38-40]. The strength of evidence across different levels was evaluated based on published guidelines (Table 3) [41].

**Table 3:** Determining evidence strength

Level of evidence	Definition
Strong	Consistent findings across multiples of high-quality research
Moderate	Consistent findings across multiples of low-quality research
Limited	Findings from one low-quality research
Conflicting	Inconsistent findings from multiple studies

Results

Literature Search Results

**Figure 2:**
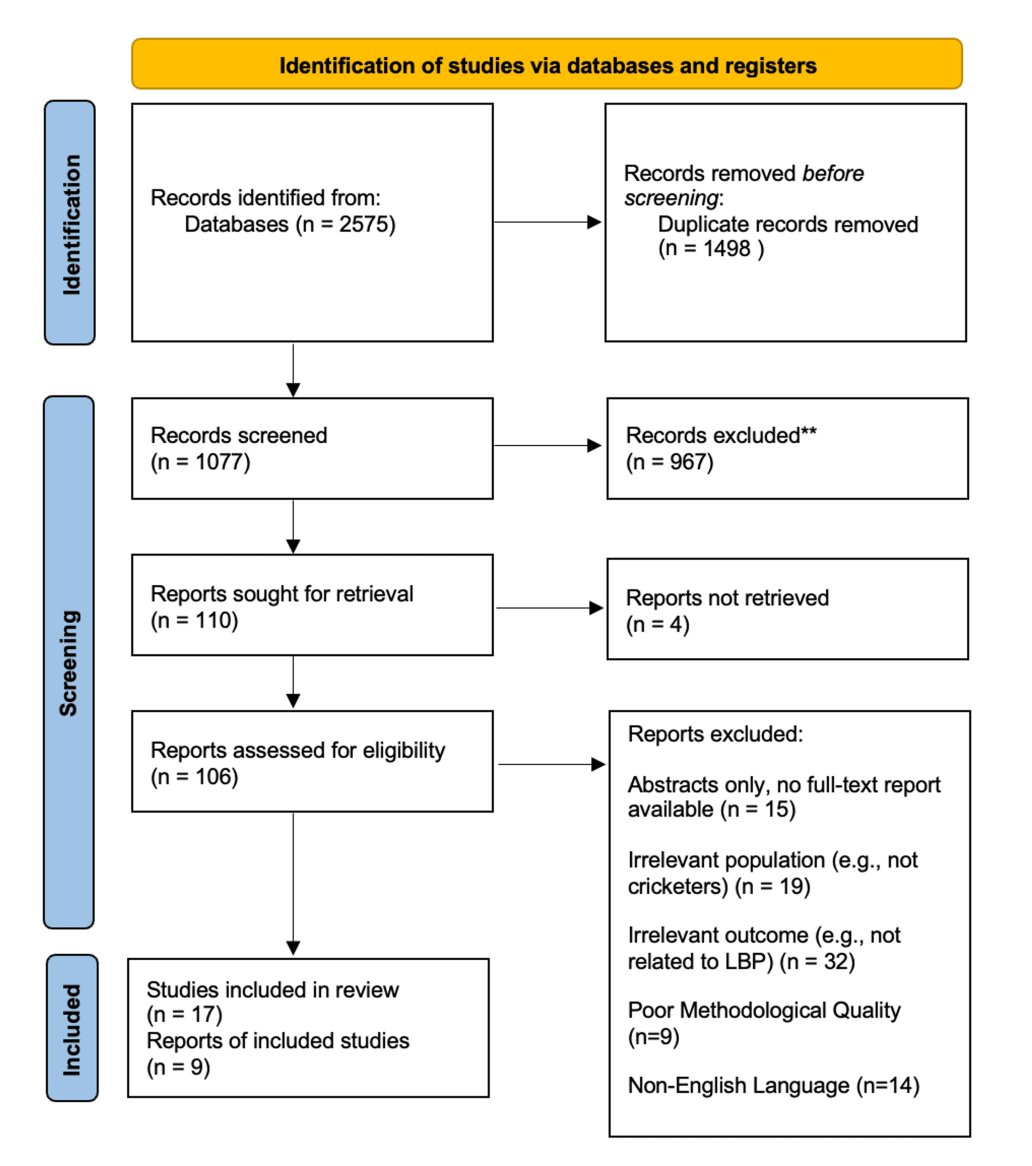
Summary of literature search following the PRISMA guidelines PRISMA: Preferred Reporting Items for Systematic Reviews and Meta-Analyses.

Figure 2 depicts the systematic process of the literature search, adhering to PRISMA guidelines. All 17 included studies met the criteria outlined in Table 1.

Methodological Characteristics

Table 4 presents an overview of the included studies; the associated quality assessment judgment is documented below. Out of the 17 studies, 11 focused on elite athletes, while six targeted sub-elite athletes (Table 4). The mean age of participants varied between 15.8 and 24.9 years [36, 42]. Only one study included female athletes within its cohort [43]. There was one interventional study that primarily investigated the reduction of LBP as its main outcome measure [44].

**Table 4:** Summary of studies included in the review Studies exploring risk factors and interventions for lower back pain (LBP) in cricketers: primary topic areas, study details, and quality assessment. LBP: lower back pain; LBSI: lumbar bone stress injury; BMO: bone marrow oedema; CSA: cross-sectional area; MRI: magnetic resonance imaging; BMD: bone mineral density; RCT: randomised controlled trial.

Primary topic area in relation to LBP	Title	Author (reference)	Study design	Participants	Outcome measure	Quality assessment
Studies exploring risk factors of LBP in cricketers						
Bowling kinematics	Injury and lumbar reposition sense in cricket pace bowlers in neutral and pace bowling-specific body positions	Olivier et al., 2014 [45]	Longitudinal study	17 male amateur fast bowlers (aged 18-26)	Injury incidence. Relationship between lumbar reposition sense (a marker of proprioception) and its association with LBP	High quality
	Lumbar load in adolescent fast bowlers: A prospective injury study	Bayne et al., 2016 [42]	Prospective cohort study	25 male elite fast bowlers (mean age 15.8 years, height 178 cm, mass 69.3 kg)	Injury incidence. Relationship between kinematics and kinanthropometrics of fast bowling action and workload with low back injury in adolescent cricketers	High quality
	Lumbar sagittal plane spinal curvature and junior-level cricket players	Hecimovich and Stomski, 2016 [43]	Cross-sectional study	33 male and 26 female amateur-level cricketers (aged 13-17)	Relationship between lumbar lordosis in junior-aged and amateur-level cricketers and lower back injury	High quality
	Pelvic and trunk mechanics and injury in cricket: A spin bowling case study	Middleton et al., 2016 [46]	Case study	1 male elite senior spin bowler	Relationship between kinematics of spin bowling action and low back injury in one cricketer	Low quality
	Biomechanical risk factors of lower back pain in cricket fast bowlers using inertial measurement units: a prospective and retrospective investigation	Senington et al., 2020 [47]	Prospective and retrospective cohort study	35 elite male fast bowlers senior (n=14; 24.1±4.3 years, 1.89±0.05 m; 89.2±4.6 kg) and junior (n=21, 16.9±0.7, 1.81±0.05, 73.0±9.2 kg)	Relationship between spinal kinematics, tibial and sacral impacts during fast bowling, among bowlers with a history of LBP (retrospective) and bowlers who developed LBP in the follow-up season (prospective)	High quality
	Cricket fast bowling technique and lumbar bone stress injury	Alway et al., 2021 [36]	Prospective study	50 elite fast bowlers (18.9±1.9 years, 83.0±8.4 kg, 1.87±0.06 m)	Injury incidence. Prospective exploration of relationship between technique and elite cricket fast bowlers who sustain an LBSI	High quality
Muscle architecture	Cricket fast bowlers without low back pain have larger quadratus (QL) lumborum asymmetry than injured bowlers	Kountouris et al., 2013 [48]	Cohort study	23 elite male fast bowlers (24.0±3.6 years, 187.3±4.9 cm, 87.3±8.3 kg)	Exploring the relationship between QL asymmetry and LBSI	High quality
	Symmetry, not asymmetry, of abdominal muscle morphology is associated with low back pain in cricket fast bowlers	Gray et al., 2016 [38]	Cross-sectional descriptive study	25 male semi-professional fast bowlers (14-18 years)	Relationship of the thickness of the abdominal muscles (transversus abdominis, olique internus, and olique externus measured with ultrasound (US) imaging) between the dominant and non-dominant sides of fast bowlers, and to compare this between fast bowlers with and without LBP	High quality
	Reduced non-dominant lumbar multifidi (LM) cross-sectional area (CSA) is a precursor of low back injury in cricket fast bowlers	Olivier et al., 2016 [49]	Prospective cohort study	26 male right-handed amateur fast bowlers (aged 21.8±1.8 years)	Injury incidence. Relationship between side-to-side symmetry of LM CSA as a potential precursor of injury in fast bowlers	High quality
MRI studies	MRI BMO precedes lumbar bone stress injury diagnosis in junior elite cricket fast bowlers	Kountouris et al., 2019 [39]	Cohort study	65 elite Australian adolescent fast bowlers (mean age 17.3)	Test the association between clinically detected BMO and BSI. Bowling workload factors that may be associated with BMO and subsequent BSI	High quality
	MRI bone marrow edema signal intensity: a reliable and valid measure of lumbar bone stress injury in elite junior fast bowlers	Sims et al., 2020 [40]	Comparative reliability and prospective validity study	65 male elite junior fast bowlers	Evaluate the reliability and validity of the assessment of LBSI using MRI BMO in elite junior fast bowlers. .	High quality
	Presence of BMO in asymptomatic elite fast bowlers: Implications for management	Taylor et al., 2021 [35]	Retrospective study	38 elite Australian fast bowlers (21.6±3.7 years)	Quantifying the intensity of BMO in elite cricketers and relating this to workload and prevalence of LBSI. Evaluate the use of MRI screening to reduce the risk of LBSI	High quality
	Activity specific areal bone mineral density (BMD) is reduced in athletes with stress fracture and requires profound recovery time: A study of LSF in elite cricket fast bowlers	Alway et al., 2022 [50]	Cross-sectional and cohort study	29 elite male fast bowlers	Relationship between lumbar BMD and fast bowlers with or without LSF. The trajectories of BMD in cricketers undergoing rehabilitation	High quality
Workload	Incidence and prevalence of LSF in English County Cricket fast bowlers, association with bowling workload and seasonal variation	Alway et al., 2019 [14]	Case–control study	368 professional English fast bowlers (24.87±6.01years)	Injury Incidence Relationship between workload and LSF	High quality
	Multiple risk factors associated with lumbar bone stress injury in youth cricket fast bowlers	Sims et al., 2021 [51]	Cohort study	222 Australian ‘high-level’ youth male fast bowlers (17.4 ± 1.1 years, range 15.1-19.7)	Relationship between LBSI and risk factors such as workload, technique, and physical preparation in youth fast bowlers	High quality
	Lumbar bone stress injuries and risk factors in adolescent cricket fast bowlers	Keylock et al., 2022 [28]	Prospective study	40 adolescent male amateur fast bowlers (aged 14-17 years)	Injury incidence association of acute workload, age, BMD, biomechanics with LBSI	High quality
Interventional studies						
Treatment	A randomised controlled study on core stability exercise programme using Swiss ball, Thera-band, and floor exercises in cricketers with low back pain	Rao et al., 2015 [44]	Randomised controlled trial (RCT)	60 male cricketers (15-35 years)	To establish the efficacy of Swiss ball (SB), Thera-band (TB), and floor exercise (FE) usage during rehabilitation in cricketers with low back pain	Low quality

Quality Assessment

Appendices present the detailed quality assessment of each study. Among the studies investigating risk factors, 15 were high quality, while one study was low quality [46]. The only interventional study included in this review was also of low quality [44]. An additional independent co-assessor conducted a quality assessment of the papers. The intraclass correlation coefficient (ICC) was 0.851, indicating good reliability in the quality assessment process [52].

Risk Factors

**Figure 3:**
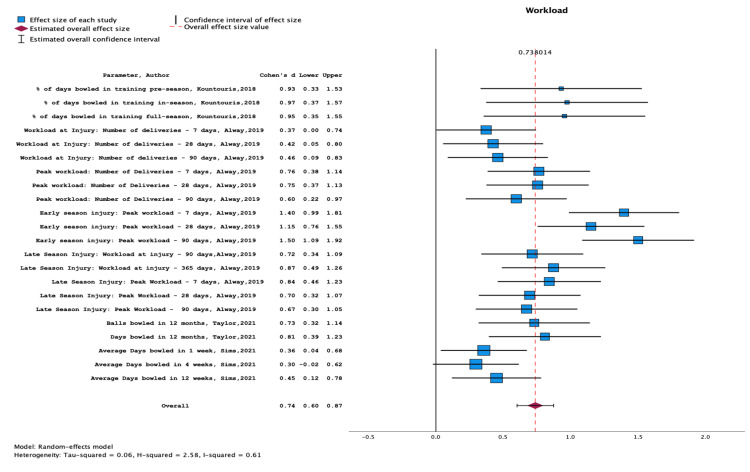
Meta-analysis of data from Kountouris et al. (2019), Alway et al. (2019), Taylor et al. (2021), and Sims et al. (2021). The results encompass variations in bowling workload and illustrate the association between increased workload and lower back injury Papers included: Kountouris et al. (2019) [39], Alway et al. (2019) [36], Taylor et al. (2021) [35] and Sims et al. (2021) [51].

**Figure 4:**
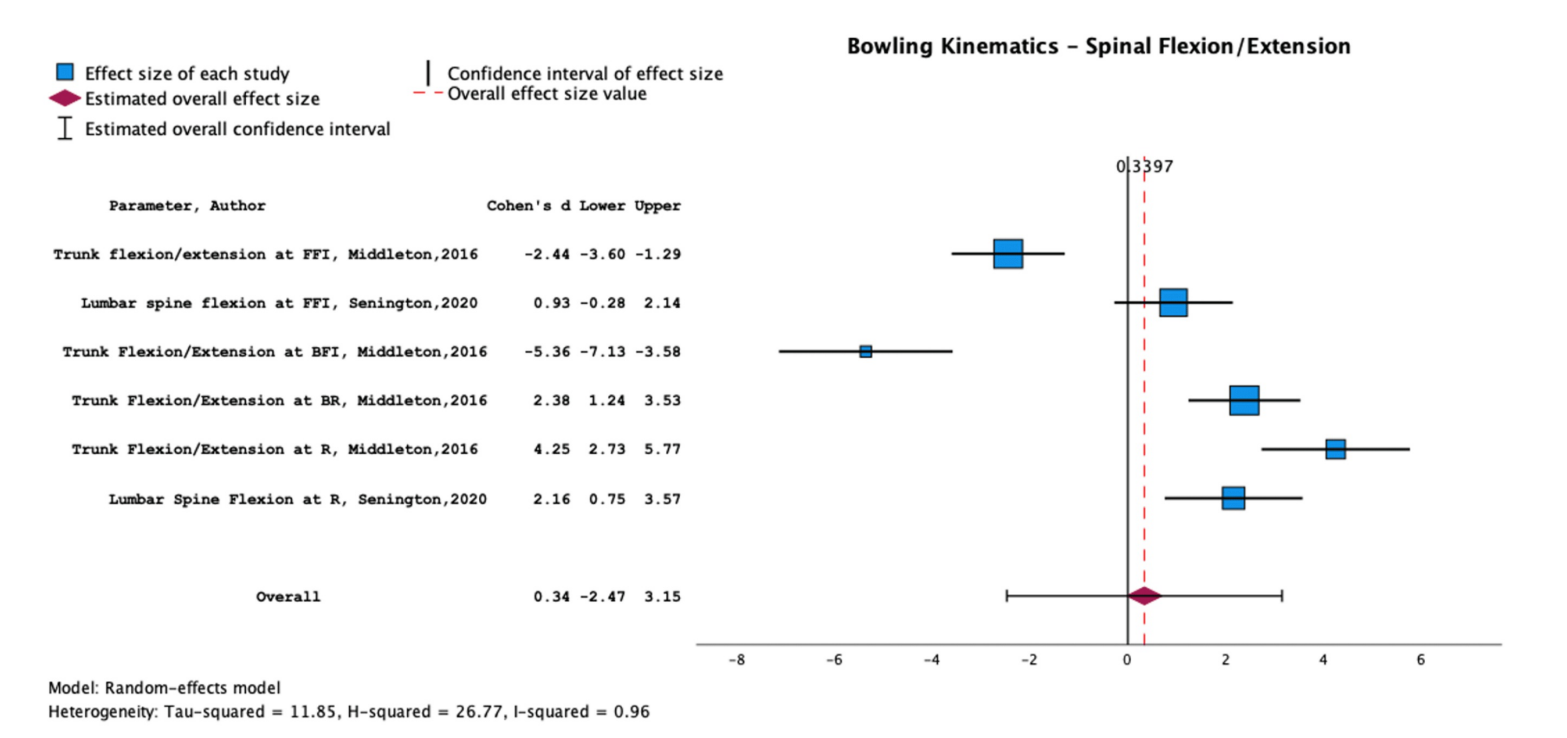
Meta-analysis of data from Middleton et al. (2016) and Senington et al. (2020). The results shown are for trunk/lumbar flexion and extension and their association with lower back injury Papers included: Middleton et al. (2016) [46] and Senington et al. (2020) [47].

**Figure 5:**
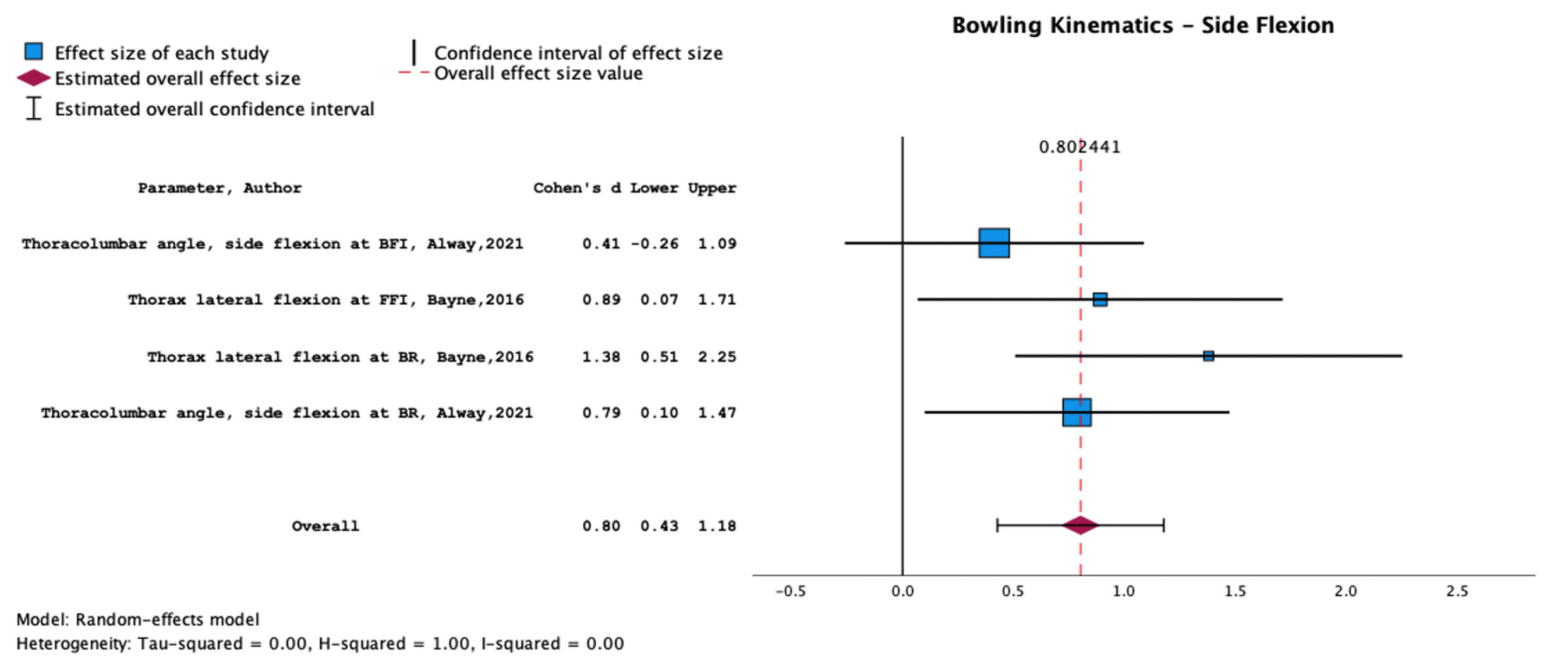
Meta-analysis of data from Alway et al. (2021) and Bayne et al. (2016) illustrating the association between lower back injury and increased side or lateral flexion Papers included: Alway et al. (2021) [50] and Bayne et al. (2016) [42].

**Figure 6:**
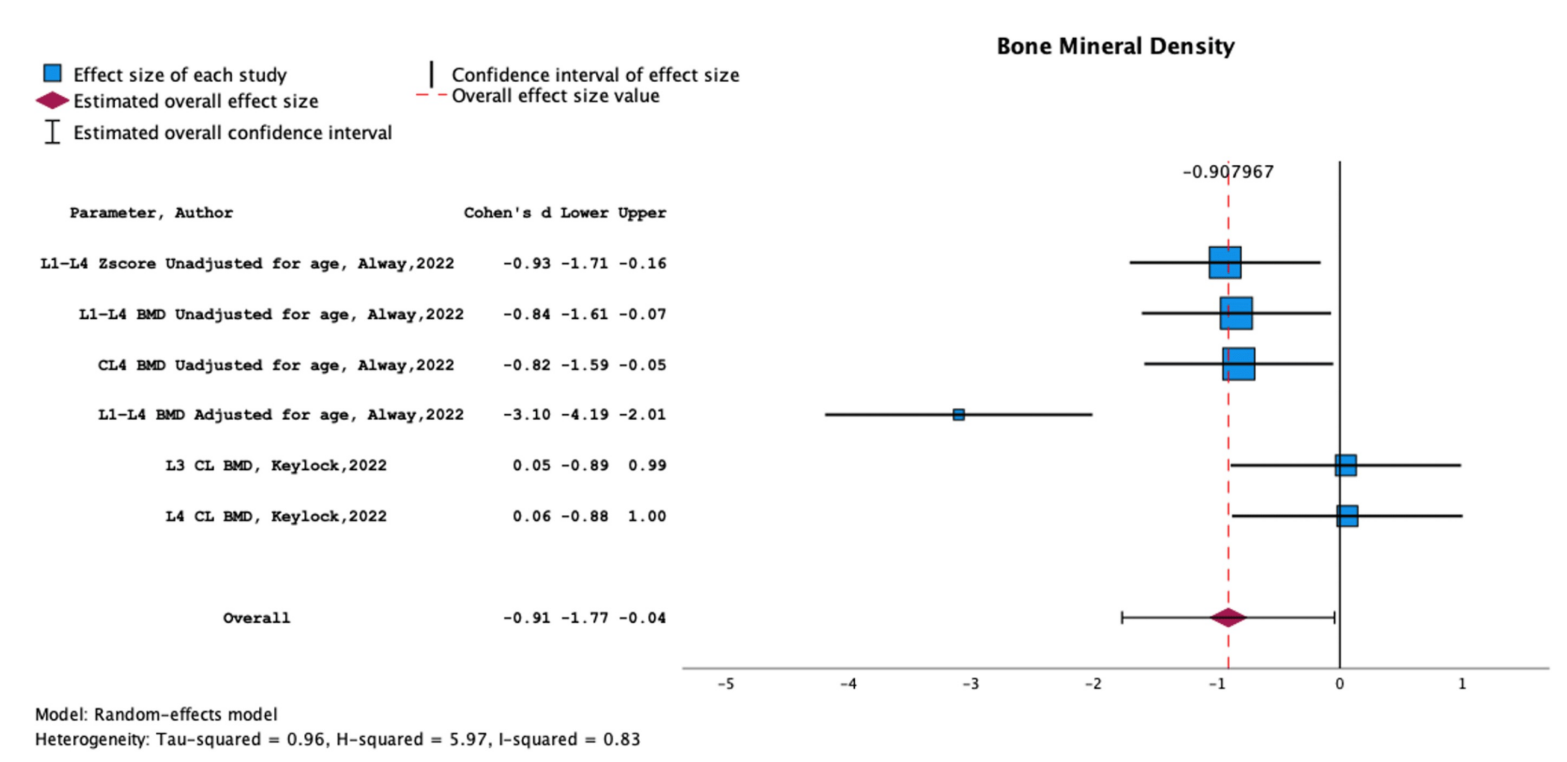
Meta-analysis of data from Alway et al. (2022) and Keylock et al. (2022) illustrating the association between lower back injury and decreased bone mineral density (BMD) Papers included: Alway et al. (2022) [50] and Keylock et al. (2022) [28].

Tables 5-8 and the corresponding Figures 3-6 present the results of meta-analyses conducted to assess the effect sizes and confidence intervals (CIs) of various risk factors for LBP. Six studies could not be included in the quantitative synthesis due to insufficient data [38, 40, 43, 45, 48, 49]. 

**Table 5:** Results of workload association with lower back injury This table summarises the relationship between workload parameters (e.g., percentage of days bowled, number of deliveries, and peak workloads) and the incidence of lower back injuries in cricketers. Data are stratified by phases of the season (pre-season, in-season, full-season) and other workload measures (e.g., early and late season injuries). Results are presented as mean ± SD for injured and non-injured groups. SD: standard deviation; FFI: front foot impact; BFI: back foot impact; BR: ball release.

Parameter	Author, year	Injured (n)	Mean ± SD	Not injured (n)	Mean ± SD
Percentage of days bowled in training pre-season	Kountouris, 2019 [39]	15	39.00 ± 23.00	50	26.00 ± 10.00
Percentage of days bowled in training in-season	Kountouris, 2019 [39]	15	41.00 ± 20.00	50	29.00 ± 9.00
Percentage of days bowled in training full-season	Kountouris, 2019 [39]	15	26.00 ± 6.00	50	21.00 ± 5.00
Workload at injury: number of deliveries - 7 days	Alway, 2019 [36]	57	128.00 ± 93.33	57	94.00 ± 88.89
Workload at Injury: number of deliveries - 28 days	Alway, 2019 [36]	57	420.67 ± 154.07	57	348.00 ± 186.67
Workload at Injury: number of deliveries - 90 days	Alway, 2019 [36]	57	1159.67 ± 457.78	57	920.33 ± 577.04
Peak workload: number of deliveries - 7 days	Alway, 2019 [36]	57	289.00 ± 61.00	57	238.00 ± 72.00
Peak workload: number of deliveries - 28 days	Alway, 2019 [36]	57	664.00 ± 173.00	57	554.00 ± 112.00
Peak workload: Number of Deliveries - 90 days	Alway, 2019 [36]	57	1410.00 ± 516.00	57	1097.00 ± 533.00
Early season injury: peak workload - 7 days	Alway, 2019 [36]	57	261.00 ± 33.00	57	189.00 ± 65.00
Early season injury: peak workload - 28 days	Alway, 2019 [36]	57	562.00 ± 71.00	57	393.00 ± 195.00
Early season injury: peak workload - 90 days	Alway, 2019 [36]	57	1013.00 ± 409.00	57	492.00 ± 271.00
Late season injury: workload at injury - number of deliveries - 90 days	Alway, 2019 [36]	57	1340.33 ± 436.30	57	975.33 ± 574.81
Late season injury: workload at injury - number of deliveries - 365 days	Alway, 2019 [36]	57	2523.00 ± 81.00	57	1974.00 ± 885.00
Late season injury: peak workload - number of deliveries - 7 days	Alway, 2019 [36]	57	305.00 ± 57.00	57	247.00 ± 79.00
Late season injury: peak workload - number of deliveries - 28 days	Alway, 2019 [36]	57	710.00 ± 185.00	57	567 ± 224.00
Late season injury: peak workload - number of deliveries - 90 days	Alway, 2019 [36]	57	1559.00 ± 541.00	57	1199.00 ± 528.00
Balls bowled in 12 months	Taylor, 2021 [35]	48	4268.67 ± 1004.44	48	3416.67 ± 1309.63
Days bowled in 12 months	Taylor, 2021 [35]	48	103.67 ± 17.78	48	88.67 ± 19.26
Average days bowled in 1 week	Sims, 2021 [51]	49	2.30 ± 0.60	170	2.00 ± 0.90
Average days bowled in 4 weeks	Sims, 2021 [51]	49	9.30 ± 2.40	170	8.20 ± 4.00
Average days bowled in 12 weeks	Sims, 2021 [51]	45	28.30 ± 7.40	170	23.60 ± 11.10

**Table 6:** Results of trunk/lumbar flexion/extension association with lower back injury This table summarises the association between trunk and lumbar flexion/extension parameters at various phases of bowling action and their correlation with lower back injury in cricketers. Data are presented as mean ± SD for injured and non-injured groups. FFI: front foot impact; BFI: back foot impact; BR: ball release; R: recovery; SD: standard deviation.

Parameter	Author, year	Injured (n)	Mean ± SD	Not injured (n)	Mean ± SD
Trunk flexion/extension at FFI	Middleton, 2016 [46]	6	-24.90 ± 0.90	18	-22.70 ± 0.90
Lumbar spine flexion at FFI	Senington, 2020 [47]	4	26.30 ± 2.60	10	20.90 ± 6.50
Trunk flexion/extension at BFI	Middleton, 2016 [46]	6	-26.60 ± 3.10	18	-16.30 ± 1.40
Trunk flexion/extension at BR	Middleton, 2016 [46]	6	3.00 ± 4.20	18	-2.83 ± 1.60
Trunk flexion/extension at R	Middleton, 2016 [46]	6	31.40 ± 4.00	18	20.70 ± 1.87
Lumbar spine flexion at R	Senington, 2020 [47]	4	50.60 ± 8.50	10	30.90 ± 9.30

**Table 7:** Results of side flexion association with lower back injury This table presents the association between thoracolumbar and thorax side flexion angles at various bowling phases and their correlation with lower back injury in cricketers. Data are presented as mean ± SD for injured and non-injured groups. FFI: front foot impact; BFI: back foot impact; BR: ball release; SD: standard deviation.

Parameter	Author, year	Injured (n)	Mean ± SD	Not injured (n)	Mean ± SD
Thoracolumbar angle, side flexion at BFI	Alway, 2021 [36]	39	182.00 ± 8.00	11	179.00 ± 3.00
Thorax lateral flexion at FFI	Bayne, 2016 [42]	12	19.90 ± 6.00	13	15.00 ± 5.00
Thorax lateral flexion at BR	Bayne, 2016 [42]	12	49.80 ± 5.90	13	40.20 ± 7.80
Thoracolumbar angle, side flexion at BR	Alway, 2021 [36]	39	163.00 ± 4.00	11	160.00 ± 3.00

**Table 8:** BMD association with lower back injury This table highlights the association between bone mineral density (BMD) and lower back injury in cricketers, presenting data for both injured and non-injured groups. Values include unadjusted and age-adjusted BMD measures across different lumbar spine levels. Data are reported as mean ± SD. L1–L4: lumbar vertebrae 1 to 4; BMD: bone mineral density; CL: cortical layer; SD: standard deviation.

Parameter	Author, year	Injured (n)	Mean ± SD	Not injured (n)	Mean ± SD
L1–L4 Z-score unadjusted for age	Alway, 2022 [50]	17	1.78 ± 1.10	12	2.77 ± 1.10
L1–L4 BMD unadjusted for age	Alway, 2022 [50]	17	1.46 ± 0.15	12	1.59 ± 0.13
CL4 BMD unadjusted for age	Alway, 2022 [50]	17	1.85 ± 0.20	12	2.07 ± 0.34
L1–L4 BMD adjusted for age	Alway, 2022 [50]	17	1.46 ± 0.04	12	1.59 ± 0.04
L3 CL BMD	Keylock, 2022 [28]	6	1.63 ± 1.16	16	1.42 ± 4.91
L3 CL BMD	Keylock, 2022 [28]	6	1.67 ± 1.05	16	1.43 ± 4.44

Workload and LBP

There was strong evidence to suggest that increased workload is associated with LBP in cricketers, with a significant overall effect size of 0.74, 95% CI (0.60, 0.87) (Figure 3). The relevant studies (Kountouris et al., 2019; Alway et al., 2019; Taylor et al., 2021; Sims et al., 2021) and their results are summarised in Table 5.

Biomechanics and Bowling Kinematics

Limited evidence suggests that trunk/lumbar flexion/extension during the bowling action is associated with LBP, with an overall effect size of 0.34 (-2.47, 3.15) (Figure 4). The studies investigating this aspect of bowling (Middleton et al., 2016; Senington et al., 2020) and their respective results are presented in Table 6.

Strong evidence indicates that side flexion is associated with LBP in cricketers, with an overall effect size of 0.80 (0.43, 1.18) (Figure 5). The results exploring this association (Alway et al., 2021; Bayne et al., 2016) are summarised in Table 7.

BMD and Injury Risk

Strong evidence indicates that lower BMD is associated with LBP, with a significant overall effect size of -0.9 (-1.77, -0.04) (Figure 6). The studies examining BMD parameters and their relationship to low back injury outcomes (Alway et al., 2022; Keylock et al., 2022) are summarised in Table 8.

Discussion

This comprehensive review updates and builds upon the foundational work of Morton et al. (2014), filling identified gaps and expanding our understanding of LBP in cricketers [16]. Our analysis included 17 studies published since the previous review, focusing on diverse risk factors such as muscle architecture, bowling biomechanics, MRI findings, and match workload. Notably, our findings underscore the strong association between increased workload and LBP development, with a significant effect size of 0.738. Moreover, our review highlights the critical role of MRI in detecting early indicators of lumbar bone stress injuries.

Impact of Workload on Injury Risk

The association between increased workload and LBP in cricketers was a prominent finding in our analysis, evidenced by a notable effect size of 0.738. This suggests a significant risk increase for LBP with heightened cricketing activity, likely important in the context of modern cricket's evolving dynamics. Notably, some studies have delineated specific workload parameters, such as the number of deliveries and bowling days, reinforcing the link between intensive playing schedules and LBP risk. Alway et al. (2019) found that bowling over 234 balls in seven days significantly increased lower bone stress injury (LBSI) risk [36]. High workload can subject the lumbar spine to repetitive loading and stress during cricket activities, leading to microdamage in vertebral structures such as intervertebral discs, facet joints, and vertebral bodies [53, 54]. This microdamage can trigger a cascade of events, including bone resorption, weakening the bone structure, and predisposing it to fractures or stress injuries [54-56]. The cumulative effect of these structural changes may manifest as symptomatic LBP, either acutely or over time [28, 51].

It was noted that younger fast bowlers exhibited a larger annual incidence and prevalence of LSF, aligning with previous findings in this population [24, 27, 55]. This susceptibility may stem from inadequate bone resilience to endure the repetitive and substantial loading associated with fast bowling, exacerbated by the biomechanical stresses imposed on the lumbar spine [57, 58]. Additionally, the delayed maturation of the lumbar spine compared to other skeletal sites could contribute to their increased risk of LSF, as full ossification may not occur until around the age of 25 [51].

However, the heterogeneity observed in our meta-analysis underscores the variability in workload measurement methods across studies, which may influence the observed associations. Workload parameters varied widely, including the number of deliveries within various timeframes (e.g. seven days, 28 days, 90 days) and the number of days bowled. For instance, Alway et al. (2019) focused on the number of balls bowled within a specific timeframe, while Sims et al. (2021) measured workload in terms of days bowled. This variability in measurement methods could contribute to discrepancies in effect sizes and hinder the generalisability of findings [36, 51].

Despite methodological differences, the practical implications of these findings for workload management in cricket are clear. In the face of modern cricket’s demanding schedules, especially with the proliferation of shorter formats like T20, managing player workload becomes pivotal. The complexity of workload impact on LBP is further exemplified by the contradictory recommendations regarding rest periods between bowling sessions. Kountouris et al. (2019) indicated through their modelling that one less day between bowling sessions could double the risk of LBSI, while Alway et al. (2019) observed a correlation between high peak workloads and injuries, especially during periods of spiking scheduling such as T20 matches, suggesting that the type of workload and its timing may influence the risk of injury [36]. This dichotomy suggests that a one-size-fits-all approach may not be feasible, and individualised workload management strategies are likely to be more effective in mitigating LBP risk among cricketers. Future research should aim to develop more nuanced guidelines that consider individual player profiles and the unique demands of different cricket formats. Additionally, potential strategies such as alterations in scheduling and limitations on bowling load for emerging fast bowlers aged between 19 and 24 years may be fruitful avenues for further exploration.

Biomechanical Considerations in Bowling

Our findings highlight a significant relationship between bowling kinematics and the occurrence of LBP in cricketers. Specifically, excessive trunk/lumbar flexion/extension and side flexion are key factors. This aligns with previous studies indicating that fast bowlers with more than a 30% deviation in these kinematic parameters are significantly more prone to developing LBP [57]. Alway et al. (2021) and Bayne et al. (2016) highlight the crucial role of efficient control of the lumbo-pelvic-femoral complex during bowling in mitigating LBP risk [36, 42]. This is in line with biomechanical principles emphasising the importance of proper kinetic chain functioning to reduce stress on the lumbar spine [58]. The notable effect size of 0.80 for side flexion association with LBP directly links certain bowling actions to spinal health, reflecting the biomechanical demands cricket bowling imposes on the body, as also indicated in studies by Morton et al. (2014), Burnett et al. (1996), and Foster et al. (1989) [16, 59, 60].

Moreover, our analysis emphasises the role of biomechanical efficiency and control in the lumbar and pelvic regions for LBP prevention, specifically identifying decreased rear hip flexion [51] at back foot contact as a key predictor for LBSI, indicative of potential strength limitations and impaired pelvic-femoral control. Alway et al. (2021) showed that bowlers who sustained an LBSI had an angle of 146° compared to 156°, which accurately classified the injury history for 76% of bowlers analysed [36, 51]. This is corroborated by findings linking increased lumbopelvic extension to higher LBSI risk, likely a result of impaired lumbopelvic control. Meta-analyses by Middleton et al. (2016) and Senington et al. (2020) support these findings, showing how different bowling techniques, such as fast and spin bowling, uniquely impact the lumbar spine through their distinct force and speed demands [46, 47].

The identification of greater lumbar extension and side flexion, along with compensatory kinematic changes, allows for targeted coaching and monitoring. Studies by Morton et al. (2014), Burnett et al. (1996), and Foster et al. (1989) have associated the "mixed action" bowling technique [16, 59, 60], involving increased extension and side flexion of the lumbar spine, with lesions of the pars interarticularis, disc injuries, and LBP. Sims et al. (2021), however, did not find a significant difference between bowling kinematics and the occurrence of LSIs [51]. This discrepancy in results may stem from the field-based approach utilised in this study, as highlighted by Alway et al. (2021) and Bayne et al. (2016) [43, 49].

The examination of biomechanical research in cricket has predominantly centred around male Caucasian subjects, prompting a call for broader inclusivity. This need for diversity is particularly pressing when considering the biomechanics of female fast bowlers, who may utilise different techniques that emphasise whole-body angular momentum and rotational movements for speed enhancement [61]. This contrast suggests a potential variation in injury risk factors and mechanisms between genders, especially concerning LBP and LSI. For instance, male bowlers are observed to have greater displacement of the centre of mass during landing phases, possibly indicating a strategy to soften landings and reduce impact forces [62]. These gender-specific biomechanical differences underscore the importance of extending research to female fast bowlers. Such studies could unveil unique risk factors and inform the development of targeted injury prevention strategies, aiming to safeguard player health and optimise performance across all demographics.

Role of Core Stability and Muscle Architecture

Previous research has been conflicting regarding the effect of increased quadratus lumborum (QL) volume and the development of LBSI [16]. Engstrom et al. (2007) indicated that asymmetries, presenting as increased QL volume on the bowling arm side, have been associated with symptomatic L4 pars lesion development in adolescents [7]. Kountouris et al. (2013) and Olivier et al. (2016) demonstrate contrasting findings, where the former found increased QL asymmetry to be less likely to result in lower back injuries, and the latter showed that injury-free fast bowlers exhibited no lumbar multifidus (LM) asymmetry [23, 48]. Gray et al. (2016) found that symmetrical abdominal muscle morphology in adolescent fast bowlers was associated with a higher likelihood of LBP, while hypertrophic adaptations of the oblique internus (OI) on the non-dominant side were absent in cricketers with LBP [50, 51]. This highlights the complexity and potential significance of muscular symmetry, particularly in the lumbar region, and its impact on LBP.

The inconsistency in findings related to the relationship between asymmetry in the QL muscle and spine injuries in cricket’s fast bowlers can partially be attributed to a scarcity of analysable images and varied approaches in muscle evaluation [50]. Moreover, the connection between the diminished thickness of the internal oblique muscle on the opposite side of the bowling arm and lower back pain becomes more complex when considering overlooked factors such as bowling workload and the muscular adjustments resulting from altered bowling practices in terms of volume and intensity [39]. Looking ahead, it is essential for studies to focus on the precise measurement of lean muscle mass and the examination of how the muscles around the trunk and pelvic area function in unison. This approach will enhance our comprehension of their influence on lower back pain and spine injuries among these athletes.

MRI and Early Detection of Stress Injuries

Morton et al. (2014) concluded that there is potential benefit in the use of MRI for screening players at high risk, ideally before they experience pre-symptomatic bone stress [16]. Advancements in MRI technology have significantly augmented our capacity to detect early indicators of LBSI, as observed in the studies by Alway et al. (2022) and Sims et al. (2020) [50, 51]. These advancements allow for earlier identification of high-risk players, potentially even before they experience pre-symptomatic bone stress. The prompt identification of symptoms, in conjunction with the detection of bone marrow oedema (BMO) and cortical breach, as opposed to cases without cortical breach [41, 62], could signify that players are already at a more advanced stage along the continuum of bone stress injury at the onset of the study [56, 63].

Advances in MRI have facilitated the objective quantification of BMO intensity, with Sims et al. (2020) proposing thresholds indicative of different stages of LBSI progression. A signal intensity exceeding 2.0 suggests early-stage asymptomatic bone stress, while levels surpassing 3.0 indicate changes associated with later symptomatic stages of LBSI [41]. Furthermore, Taylor et al. (2021) and Kountouris et al. (2019) explored the predictive value of BMO intensity in identifying high-risk individuals prone to LBSI development [35, 39]. Taylor et al. (2021) observed a significantly elevated risk of LBSI within 12 months among bowlers with BMO intensity exceeding 2.0, emphasising the prognostic significance of MRI-detected BMO. However, Kountouris et al. (2019) reported a higher relative risk among junior elite cricketers with BMO, highlighting the multifactorial nature of LBSI beyond workload considerations [39].

Thorough reliability assessments conducted by Taylor et al. (2021) and Sims et al. (2020) reaffirm the validity of MRI-based BMO measurements. These findings underscore the clinical utility of BMO monitoring as a diagnostic tool for identifying athletes at risk of LBSI. Moreover, Kountouris et al. (2019) advocate for a structured de-loading period following the detection of asymptomatic BMO to prevent progression to LSF, thereby emphasising the importance of early intervention strategies guided by MRI findings [39, 40, 41].

The findings from muscle architecture and MRI studies provide valuable insights into the early screening and prevention of LBP in cricketers. They suggest that incorporating assessments of muscle symmetry and regular MRI screenings into routine health checks for cricketers could be beneficial. This proactive approach, informed by the latest research, would not only aid in early intervention but also help in tailoring individualised training and rehabilitation programmes, thereby enhancing the long-term health and performance of cricketers.

Implications of Bone Mineral Density for Injury Prevention

MRI findings reveal an increase in bone marrow oedema, indicating early lumbar stress injury. This highlights the importance of assessing bone health, as inadequate BMD may predispose cricketers, especially younger athletes, to more severe stress injuries over time.

Our study has revealed a significant association between lower BMD and LBP in cricketers, evidenced by a substantial overall effect size of -0.9. This finding, as supported by the studies of Alway et al. (2022) and Keylock et al. (2022), indicates that decreased BMD, particularly in the lumbar region, is a critical factor contributing to LBP in athletes​​. However, causality cannot be definitively determined through cross-sectional studies [28, 50].

Alway et al. (2022) suggest that among fast bowlers, there is a decline in BMD within the lumbar vertebrae approximately 21 to 24 weeks following an LSF, indicating a delayed recuperation of BMD after the injury event. This delayed recuperation may potentially contribute to the recurrence of LBP [50]. The decrease in BMD might not only be a result of mechanical stress but also associated with physiological factors such as nutritional deficits or hormonal imbalances [56, 63].

The significance of this finding lies in its potential application in athlete health monitoring and injury prevention strategies. Regular BMD assessments could become a standard part of health screenings for cricketers, particularly those in high-stress roles like fast bowling. Early detection of decreased BMD could lead to timely interventions, potentially preventing the progression to more severe injuries or chronic LBP.

Effectiveness of Interventions for LBP

A randomised controlled trial (RCT) conducted by Rao et al. (2015) offers preliminary insights into the potential benefits of core-strengthening exercises for cricketers suffering from LBP [44]. The study evaluated four different four-week core exercise programmes (Swiss ball, Thera-band, and floor exercise) involving 60 male cricketers with LBP and revealed a significant amelioration in pain severity among participants, particularly evident in those subjected to the Thera-band programme relative to the control group. This observation underscores the significance of targeting core musculature activation, common to all three exercise modalities, as a mechanism for segmental enhancement, consequently reducing stress and dysfunction within the lumbar region [64-66]. However, the study's limitations, including inadequate participant information, a small sample size, and the brief duration of the intervention, constrain the strength and applicability of its conclusions. These findings, despite their limitations, underscore the potential value of core-strengthening exercises in managing LBP in cricketers [44, 45].

Prior intervention research demonstrated the effectiveness of a 13-week abdominal stabilisation training regimen in enhancing abdominal fascia slide and reducing asymmetry of multifidus muscles, with potential implications for lowering LBP [64-66]. Crucially, analogous programmes implemented in high-risk athletic populations, such as golfers, have also shown promise in mitigating LBP [67]. Future studies should aim for larger sample sizes, longer intervention durations, and more comprehensive participant information to validate and extend these preliminary findings.

Limitations of the Current Study

In interpreting the findings of this study, it is important to address certain limitations that may influence the scope and reliability of our conclusions. Firstly, while the level of agreement in assessing methodological bias using a modified version of Downs and Black’s method was generally high, the final judgments were somewhat subjective. This subjectivity introduces a potential source of bias in our analysis. Furthermore, the selection criteria for studies may have introduced bias, as factors such as geographic location, player demographics, and levels of play could influence the generalisability of our findings. The heterogeneity among the included studies in terms of methodology, participant characteristics, and assessment criteria also poses challenges in synthesising and interpreting the results uniformly, which may limit the applicability of our findings across diverse cricket player populations and contexts. Additionally, the dynamic nature of cricket, characterised by evolving playing formats and training techniques, underscores the need for continued research to ensure the relevance and applicability of these findings amidst such changes.

Key Areas for Future Research

Several critical themes emerge from the updated synthesis of the literature on LBP in cricketers. Firstly, while the current body of research provides valuable insights into risk factors such as workload, bowling kinematics, muscle architecture, and MRI findings, there remains a need for further investigation to address gaps and inconsistencies in the literature.

One key area for future research lies in the evaluation of interventions for the treatment and prevention of LBP in cricketers. Despite the identification of promising interventions such as core-strengthening exercises, the limited number of interventional studies, particularly RCTs, underscores the need for more robust research in this area. Additionally, studies with larger sample sizes and longer intervention durations are needed to better understand the effectiveness of these interventions in the context of LBP management. Furthermore, existing rehabilitation and return-to-play guidelines require scientific validation to ensure their efficacy and implementation [66, 67]. The role of abdominal muscle asymmetry in LBP among cricketers remains unclear, with conflicting evidence from existing studies. Future research should aim to clarify the relationship between abdominal muscle architecture, lumbo-pelvic-femoral control, and the occurrence of LBP, potentially through prospective cohort studies or biomechanical analyses.

Another crucial avenue for future investigation lies in the application of MRI as a screening tool for LBP in cricketers. While recent studies have shown promising results in identifying early indicators of bone stress and decreased BMD through MRI, further research is needed to establish standardised protocols for screening and interpretation of MRI findings. Additionally, there is a clear need for research that encompasses a broader demographic, including female cricketers. As the popularity of women’s cricket continues to grow, understanding the nuances of LBP in this group becomes increasingly important. Research in this area should consider gender-specific factors that might influence the incidence, manifestation, and treatment of LBP.

Overall, the synthesis of current research underscores the multifaceted nature of LBP in cricketers and emphasises the necessity for continued investigation into risk factors, interventions, and screening methods. Addressing these knowledge gaps through high-quality research will not only enhance our understanding of LBP in cricket but also inform the development of more effective prevention and management strategies for players at all levels of the sport. Additionally, concerted efforts from national and international cricketing bodies to commission and resource significant prospective research are essential to advance the understanding, prevention, and management of this prevalent sport-specific issue.

## Conclusions

This review provides a thorough update on the literature regarding LBP in cricketers, offering key insights for medical and coaching staff in managing this condition. We have identified LBP as a multifactorial issue, strongly associated with factors including increased workload, lumbo-pelvic-femoral control issues, BMD changes, and MRI findings such as BMO.

However, our analysis reveals critical research gaps. The unclear relationship between abdominal muscle asymmetry and LBP, along with the scarcity of high-quality interventional studies, particularly for core-strengthening exercises, highlights areas needing further exploration. Additionally, the lack of focused research on female cricketers is a significant omission, given their increasing participation in the sport. Future studies should prioritise exploring under-researched areas, especially concerning female cricketers, and enhancing the quality of interventional research to develop more effective LBP management strategies for the cricket community.

## Appendices

**Table 9:** Quality assessment scores for the interventional study using Downs and Black tool This table presents the quality assessment scores for the interventional study conducted by Rao et al. (2015) [44], using the modified Downs and Black scoring tool [33] for studies researching risk factors or interventions. The scoring system, as outlined by Morton et al. (2014) [16], considers a score >20/27 as high quality. Individual assessment criteria are listed alongside the binary scoring (1 for "yes," 0 for "no"). Downs and Black: A checklist for measuring the quality of both randomized and non-randomized studies of healthcare interventions.

Study	Rao et al. (2015) [44]
Scoring	Modified Downs and Black for studies researching risk factors (>20/27 is considered high quality) (Morton et al. 2014)
Overall score	19/27
Is the hypothesis/aim/objective of the study clearly described?	1
Are the main outcomes to be measured clearly described in the Introduction or Methods section?	1
Are the characteristics of the patients included in the study clearly described?	0
Are the interventions of interest clearly described?	1
Are the distributions of principal confounders in each group of subjects to be compared clearly described?	0
Are the main findings of the study clearly described?	1
Does the study provide estimates of the random variability in the data for the main outcomes?	1
Have all important adverse events that may be a consequence of the intervention been reported?	0
Have the characteristics of patients lost to follow-up been described?	1
Have actual probability values been reported (e.g. 0.035 rather than <0.05) for the main outcomes except where the probability value is less than 0.001?	1
Were the subjects asked to participate in the study representative of the entire population from which they were recruited?	0
Were those subjects who were prepared to participate representative of the entire population from which they were recruited?	1
Were the staff, places, and facilities where the patients were treated, representative of the treatment the majority of patients receive?	1
Was an attempt made to blind study subjects to the intervention they have received?	1
Was an attempt made to blind those measuring the main outcomes of the intervention?	0
If any of the results of the study were based on “data dredging”, was this made clear?	1
In trials and cohort studies, do the analyses adjust for different lengths of follow-up of patients, or in case-control studies, is the time period between the intervention and outcome the same for cases and controls?	1
Were the statistical tests used to assess the main outcomes appropriate?	1
Was compliance with the intervention/s reliable?	1
Were the main outcome measures used accurate (valid and reliable)?	1
Were the patients in different intervention groups (trials and cohort studies) or were the cases and controls (case-control studies) recruited from the same population?	1
Were study subjects in different intervention groups (trials and cohort studies) or were the cases and controls (case-control studies) recruited over the same period of time?	1
Were study subjects randomized to intervention groups?	1
Was the randomized intervention assignment concealed from both patients and health care staff until recruitment was complete and irrevocable?	0
Was there adequate adjustment for confounding in the analyses from which the main findings were drawn?	0
Were losses of patients to follow-up taken into account?	1
Did the study have sufficient power to detect a clinically important effect where the probability value for a difference being due to chance is less than 5%?	0

**Table 10:** Quality assessment scores for risk factors using modified Downs and Black tool (Barton et al., 2009, Prince et al., 2008) This table presents the quality assessment scores for studies investigating risk factors associated with lower back pain (LBP) in cricketers, using the modified Downs and Black tool. The scoring system, as described by Morton et al. (2014), rates studies on a 16-point scale, with scores >10 considered high quality. Individual assessment criteria include study design, population description, outcome clarity, statistical testing, and confounder adjustment. LBP: lower back pain. Downs and Black: A checklist for assessing the methodological quality of healthcare studies.

Studies	Olivier et al., 2014 [45]	Bayne et al., 2016 [42]	Hecimovich and Stomski, 2016 [43]	Middleton et al., 2016 [46]	Senington et al., 2020 [47]	Alway et al., 2021 [50]	Kountouris et al., 2013 [48]	Gray et al., 2016 [38]	Olivier et al., 2016 [25]	Kountouris et al., 2019 [39]	Sims et al., 2020 [40]	Taylor et al., 2021 [35]	Alway et al., 2022 [50]	Alway et al., 2019 [36]	Sims et al., 2021 [51]	Keylock et al., 2022 [28]	
Scoring	Modified Downs and Black for studies researching risk factors (>10/16 is considered high quality) (Morton et al. 2014)																
Overall score	11/16	13/16	10/16	8/16	11/16	11/16	11/16	12/16	12/16	13/16	12/16	13/16	12/16	12/16	12/16	11/16	
1. Is the hypothesis/aim/objective of the study clearly described?	1	1	1	1	1	1	1	1	1	1	1	1	1	1	1	1	
2. Are the main outcomes to be measured clearly described in the Introduction or Methods section?	1	1	1	1	1	1	1	1	1	1	1	1	1	1	1	1	
3. Are the characteristics of the patients included in the study clearly described?	1	1	1	0	1	1	1	1	1	1	1	1	1	1	1	1	
4. Are the interventions of interest clearly described?																	
5. Are the distributions of principal confounders in each group of subjects to be compared clearly described?	1	1	0	0	0	0	1	1	1	1	0	1	1	1	1	0	
6. Are the main findings of the study clearly described?	1	1	1	1	1	1	1	1	1	1	1	1	1	1	1	1	
7. Does the study provide estimates of the random variability in the data for the main outcomes?	1	1	1	1	1	1	0	1	1	1	1	1	1	1	1	1	
8. Have all important adverse events that may be a consequence of the intervention been reported?																	
9. Have the characteristics of patients lost to follow-up been described?																	
10. Have actual probability values been reported (e.g. 0.035 rather than <0.05) for the main outcomes except where the probability value is less than 0.001?	1	1	1	0	1	1	1	1	1	1	1	1	1	1	1	1	
11. Were the subjects asked to participate in the study representative of the entire population from which they were recruited?	0	0	0	0	0	0	0	0	0	0	0	0	0	0	0	0	
12. Were those subjects who were prepared to participate representative of the entire population from which they were recruited?	0	1	1	0	1	1	1	1	1	1	1	1	1	1	1	1	
13. Were the staff, places, and facilities where the patients were treated, representative of the treatment the majority of patients receive?																	
14. Was an attempt made to blind study subjects to the intervention they have received?																	
15. Was an attempt made to blind those measuring the main outcomes of the intervention?	0	0	0	0	0	0	0	0	0	1	1	1	0	0	0	0	
16. If any of the results of the study were based on “data dredging”, was this made clear?	1	1	1	1	1	1	1	1	1	1	1	1	1	1	1	1	
17. In trials and cohort studies, do the analyses adjust for different lengths of follow-up of patients, or in case-control studies, is the time period between the intervention and outcome the same for cases and controls?																	
18. Were the statistical tests used to assess the main outcomes appropriate?	1	1	1	1	1	1	1	1	1	1	1	1	1	1	1	1	
19. Was compliance with the intervention/s reliable?																	
20. Were the main outcome measures used accurate (valid and reliable)?	1	1	1	1	1	1	1	1	1	1	1	1	1	1	1	1	
21. Were the patients in different intervention groups (trials and cohort studies) or were the cases and controls (case-control studies) recruited from the same population?	1	1	0	1	1	1	1	1	1	1	1	1	1	1	1	1	
22. Were study subjects in different intervention groups (trials and cohort studies) or were the cases and controls (case-control studies) recruited over the same period of time?																	
23. Were study subjects randomized to intervention groups?																	
24. Was the randomized intervention assignment concealed from both patients and health care staff until recruitment was complete and irrevocable?																	
25. Was there adequate adjustment for confounding in the analyses from which the main findings were drawn?	0	1	0	0	0	0	0	0	0	0	0	0	0	0	0	0	
26. Were losses of patients to follow-up taken into account?																	
27. Did the study have sufficient power to detect a clinically important effect where the probability value for a difference being due to chance is less than 5%?																	
